# microRNA Expression in Sentinel Nodes from Progressing Melanoma Patients Identifies Networks Associated with Dysfunctional Immune Response

**DOI:** 10.3390/genes7120124

**Published:** 2016-12-14

**Authors:** Viviana Vallacchi, Chiara Camisaschi, Matteo Dugo, Elisabetta Vergani, Paola Deho, Ambra Gualeni, Veronica Huber, Annunziata Gloghini, Andrea Maurichi, Mario Santinami, Marialuisa Sensi, Chiara Castelli, Licia Rivoltini, Monica Rodolfo

**Affiliations:** 1 Immunotherapy Unit, Department of Experimental Oncology and Molecular Medicine, Fondazione IRCCS Istituto Nazionale dei Tumori, Milan 20133, Italy; viviana.vallacchi@istitutotumori.mi.it (V.V.); chiara.camisaschi@istitutotumori.mi.it (C.C.); elisabetta.vergani@istitutotumori.mi.it (E.V.); paola.deho@istitutotumori.mi.it (P.D.); veronica.huber@istitutotumori.mi.it (V.H.); chiara.castelli@istitutotumori.mi.it (C.C.); licia.rivoltini@istitutotumori.mi.it (L.R.); 2 Functional Genomics and Bioinformatics, Department of Experimental Oncology and Molecular Medicine, Fondazione IRCCS Istituto Nazionale dei Tumori, Milan 20133, Italy; matteo.dugo@istitutotumori.mi.it (M.D.); marialuisa.sensi@istitutotumori.mi.it (M.S.); 3 Molecular Pathology Unit, Department of Pathology, Fondazione IRCCS Istituto Nazionale dei Tumori, Milan 20133, Italy; ambra.gualeni@istitutotumori.mi.it (A.G.); annunziata.gloghini@istitutotumori.mi.it (A.G.); 4 Melanoma and Sarcoma Unit, Department of Surgery, Fondazione IRCCS Istituto Nazionale dei Tumori, Milan 20133, Italy; andrea.maurichi@istitutotumori.mi.it (A.M.); mario.santinami@istitutotumori.mi.it (M.S.)

**Keywords:** microRNA, sentinel node biopsy, melanoma, immunosuppression, CD30

## Abstract

Sentinel node biopsy (SNB) is a main staging biomarker in melanoma and is the first lymph node to drain the tumor, thus representing the immunological site where anti-tumor immune dysfunction is established and where potential prognostic immune markers can be identified. Here we analyzed microRNA (miR) profiles in archival tumor-positive SNBs derived from melanoma patients with different outcomes and performed an integrated analysis of transcriptional data to identify deregulated immune signaling networks. Twenty-six miRs were differentially expressed in melanoma-positive SNB samples between patients with disease progression and non-progressing patients, the majority being previously reported in the regulation of immune responses. A significant variation in miR expression levels was confirmed in an independent set of SNB samples. Integrated information from genome-wide transcriptional profiles and in vitro assessment in immune cells led to the identification of miRs associated with the regulation of the TNF receptor superfamily member 8 (*TNFRSF8*) gene encoding the CD30 receptor, a marker increased in lymphocytes of melanoma patients with progressive disease. These findings indicate that miRs are involved in the regulation of pathways leading to immune dysfunction in the sentinel node and may provide valuable markers for developing prognostic molecular signatures for the identification of stage III melanoma patients at risk of recurrence.

## 1. Introduction

In cutaneous melanoma, sentinel node biopsy (SNB) is the standard clinical practice for intermediate thickness melanoma, providing important staging information for stratifying metastatic risk and for programming adjuvant treatments [1]. SNB positivity upstages a patient to stage III disease, characterized by a highly variable five-year survival rate (78%, 59% and 40% for substages A, B and C, respectively) and defined by the extent of lymph node involvement and the characteristics of the primary tumor [2]. New prognostic markers able to improve the identification of patients at high risk for disease recurrence are critically needed even more today now that effective therapies for metastatic melanoma have been developed. Beyond its value as a staging biomarker, the sentinel node directly drains the tumor and represents the first immunological site where dysfunctions of anti-tumor immune response are established and where potential prognostic markers can be identified.

The microRNA (miR) family of non-coding RNAs is endowed with important regulatory roles in the immune system. miRs are active during hematopoietic development and lineage differentiation, and regulate several aspects of the immune response, from inflammation to both innate and adaptative immunity, by targeting genes involved in immune cell development, proliferation, differentiation and function [3,4,5]. In tumors, miRs have been shown to condition the tumor microenvironment to a protumorigenic milieu by modulating secretion of cytokines and the expression of costimulatory molecules [6,7].

We have recently observed that significant variation in immune response-related gene expression characterizes melanoma-positive SNB from patients with disease progression [8]. Our results support the view that tumor-induced immune suppression occurring in sentinel nodes anticipates and contributes to subsequent metastatic recurrence [9], thus providing immune-related markers with prognostic implications [10]. To evaluate whether miRs can be included on the list of differentially expressed transcripts associated with disease progression in SNB, and to assess if they are involved in regulating the molecular pathways of suppressed immunity, here we analyzed miR profiles in tumor-positive SNB from patients with different outcomes. Testing the same set of archival SNB samples previously studied for gene expression allowed us to perform integrated analysis of information from genome-wide transcriptional profiles and miR profiles using bioinformatic tools, in order to investigate whether specific miR profiles may be associated with defined pathways of immune dysfunction. The findings here reported support the involvement of miRs in the modulation of sentinel node immune status and indicate that miRs may represent useful markers for the definition of prognostic molecular signatures for stage III melanoma patients.

## 2. Materials and Methods

### 2.1. Clinical Samples

SNB from 39 melanoma patients with relapse or not within five years of follow-up were studied. Formalin-fixed paraffin-embedded (FFPE) SNB tissues were selected as previously reported [8]. The diagnostic histological data and the clinical data for the studied cases are reported in Supplementary material Table S1. Fresh melanoma metastases specimens were collected in RNAlater Stabilization Solution (Ambion, Thermo Fisher Scientific, Waltham, MA, USA) and stored at −20 °C until use. The study was reviewed and approved by the Institutional Review Board and the Independent Ethics Committee, and written informed consent was obtained from the patients.

### 2.2. Microarray Profiling and Data Analysis

miR profiles were obtained using Agilent Human miRNA miRBase 16 microarrays Agilent Human miRNA miRBase 16 microarrays . Images were scanned with Agilent SureScan scanner and raw data were collected using Agilent′s Feature Extraction software v10.7 (Agilent Technologies, Santa Clara, CA, USA). Raw data were preprocessed using the limma package [11]. Briefly, after background correction with the normexp method, raw data were normalized with quantile method and log2 transformed. Spots with intensity greater than 10% of the 95th percentile of negative controls in at least two samples were included in further analyses. Intensities of replicated probes were summarized using the mean and for each set of probes mapping on the same miR the probe with highest mean expression was selected. Differentially expressed miRs were identified using the limma package. Multiple-testing correction was performed using the Benjamini-Hochberg false discovery rate (FDR) [12]. Genes with FDR < 0.1 and absolute fold-change ≥ 1.2 were considered significant. The data were deposited in the Gene Expression Omnibus repository (GSE88727). Ingenuity Pathway Analysis (IPA, Qiagen, Redwood City, CA, USA, www.qiagen.com/ingenuity) was used to identify gene networks and biological pathways regulated by miRs modulated in SNB from progressing patients. In silico prediction of miR targets was performed with the algorithms microT-CDS, the microRNA.org database (http://www.microrna.org/microrna/home.do) and TargetScan v6.2.

### 2.3. RNA Extraction and Quantitative Real-Time PCR (qRT-PCR) Analysis

RNA was extracted from FFPE tissue sections using the RecoverAll Total Nucleic Acid Isolation Kit (Ambion, Foster City, CA, USA), from melanoma specimens using the mirVana miRNA Isolation Kit (Ambion) and from in vitro stimulated lymphocytes using the NucleoSpin miRNA kit (Macherey-Nagel, Duren, Germany). Gene and miR expression levels were evaluated by qRT-PCR using Thermo Fisher Scientific reagents (Waltham, MA, USA) with the exception of miR-574-5p. microRNA Reverse Transcription Kit (Thermo Fisher), with the associated RT primer pool, or High-Capacity cDNA Archive Kit (Thermo Fisher) were used to retrotranscribe miRs and mRNAs, respectively; TaqMan PreAmp Master Mix (Thermo Fisher) with the associated pre-amplification primer pool was used for pre-amplifications. miR-574-5p expression was assessed by Qiagen assays (Qiagen, Hilden, Germany), after retrotranscription by miScript II RT Kit (Thermo Fisher) and pre-amplification by miScript PreAMP PCR Kit (Thermo Fisher) using as internal reference U6 snRNA (Qiagen, Hilden, Germany). *TNFRSF8*, TNF Receptor Superfamily Member 8 (Hs00174277_m1) and Beta Actin (4326315E) TaqMan Gene Expression assays were used. All qRT-PCRs were carried out in triplicate and run on the ABI Prism 7900HT instrument. Results were analyzed using the SDS software version 2.2.2 (Thermo Fisher Waltham, MA, USA). Relative quantification was determined using 2^−∆Ct^ or 2^−∆∆Ct^ method, and statistical analysis was performed by applying the non-parametric Mann-Whitney *U*-test or unpaired *t*-test as indicated in the figure legends.

### 2.4. miR In Situ Hybridization (ISH) and Tissue Immunostaining

miR ISH was performed as previously described [13]. Tumor sections were hybridized with double-DIG-LNA probes for miR-574-5p (38674-15) and miR-1246 (21239-15), and positive and negative control (Exiqon, Vedbaek, Denmark). Immunohistochemistry was carried out using an automated immunostainer (BenchMark Ultra, Ventana Medical Systems, Tucson, AZ, USA) with anti-CD3 (A0452) and anti-CD20 antibodies (M0755) purchased from Dako (Glostrup, Denmark).

### 2.5. In vitro Lymphocyte Cultures and Fluorescence Activated Cell Sorting (FACS) Analysis

Peripheral blood mononuclear cells (PBMCs) were isolated using Ficoll-PaqueTM PLUS density gradient centrifugation (GE Healthcare Bio-Sciences, Uppsala, Sweden) from blood samples of donors collected upon informed consent. CD4^+^CD25^−^ T cells and CD4^+^CD25^+^ regulatory T cell (Tregs) were purified using the CD4^+^CD25^+^ regulatory T Cell Isolation Kit (Miltenyi Biotec, Bergisch Gladbach, Germany). Tregs were expanded in vitro as previously reported [14]. Naïve CD4^+^CD25^−^ T cells and expanded Tregs were cultured as previously described [14]. Cells were activated for 72 h in vitro using IL-2 (50 U/mL) (Proleukin-Chiron, Amsterdam, The Netherlands) and anti-CD3/CD28 microbeads (0.5 µL/mL) (Dynabeads, Thermo Fisher Scientific). Simultaneously, transient transfection of CD4^+^CD25^−^ T cells was carried out by using HiPerFect lipid (Qiagen). miR-30c-5p, miR-23a-3p and miR-4299 were overexpressed using a pool of mirVana miRNA mimics (MC17563, MC10644, MC11060), and mirVana miRNA Mimic Negative Control (4464058) was used as scrambled control (Ambion). PBMCs were cultured for 48 h in RPMI (Lonza, Basel, Switzerland) supplemented with 10% human serum in the presence or absence of melanoma-conditioned medium obtained from LM12 melanoma cell line upon 72-h culture in RPMI supplemented with 10% fetal bovine serum. FACS analysis was carried out with PE-conjugated anti-CD30 mAb or isotype control. For PBMC analysis the following anti-human mAbs were used: Percp5.5 conjugated anti-CD3, FITC anti-CD14, PE anti-CD30 and PeCy7 anti-HLA-DR (BD Biosciences, San Jose, CA, USA). Live cells were identified using a Live/Dead Fixable Dead Cell Stain Kit (Invitrogen, Carlsbad, CA, USA). Data acquisition was performed using Gallios flow cytometer (Beckman Coulter, Brea, CA, USA) and data analysis was performed with FlowJo Cytometry Analysis software (FloJow, Ashland, OR, USA). Two independent experiments were performed with similar results, and representative results are presented.

## 3. Results

### 3.1. Differential miR Expression Characterizes SNB in Patients with Progressive Disease

**Figure 1:**
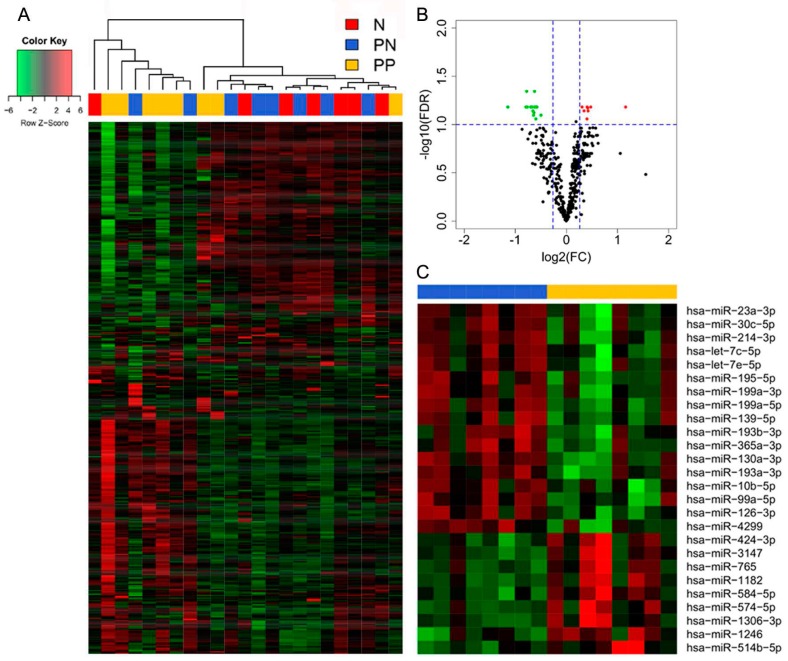
microRNA (miR) profiles of sentinel node biopsy (SNB) distinguish patients with progressing disease. (**A**) Unsupervised hierarchical clustering of miR profiles for the 24 SNB samples shows a segregation of patients with poor prognosis (PP) from those with good prognosis (PN and N); (**B**) Volcano plot from the comparison of PP and PN samples. The *x*-axis represents log2 fold-change and the *y*-axis represents false discovery rate (−log10). The 9 up-regulated genes (false discovery rate, FDR < 0.1 and log2-FC > 1.2) are highlighted in red while the 17 downregulated genes (FDR < 0.1 and log2-FC < −1.2) in green; (**C**) Heatmap showing the expression pattern of the 26 differentially expressed miRs in PP and PN classes, also listed in Table 1. Please define separately.

miR profiling analysis was carried out in a set of 24 archival SNB samples including tumor-positive SNB samples from patients progressing (PP) or not (PN) within five years of follow-up and tumor-negative (N) SNB samples from non-relapsing patients (Supplementary material Table S1). Unsupervised cluster analysis showed that most tumor-positive SNB samples from patients with progressing disease displayed miR profiles different from those of SNB samples of N and PN groups (Figure 1A), indicating that, among tumor-positive SNB, those from patients with poor prognosis are characterized by different miR patterns. By class comparison between the tumor-positive SNB samples from patients with different clinical outcome, 26 differentially expressed miRs were identified in SNB from progressors (FDR < 0.1, fold change (FC) > |1.2|). Of these, 9 displayed upregulation and 17 downregulation (Figure 1B,C, Table 1).

The list of the identified miRs was evaluated for information on their involvement in immune cells in published studies. According to the analysis, 20 out of 26 miRs have documented functions in immune cell regulation, and specifically in T cell activation (miR-1246, 214-3p, 130a-3p, 23a-3p, 199-5p, 126-3p, 193a-3p, 365-3p, 139-5p), myeloid cell differentiation and function (574-5p, 424-3p, 584-5p, 199a-3p, 195-5p, 30c-5p, let7c-5p, 23a-3p, let7e-5p, 10b-5p) and B cell differentiation (99a-5p) (Table 2). These results suggest that miR profiles of the tumor-positive node in patients with progressive disease reflect broad immune cell regulatory mechanisms, mostly involving T cells and monocytes. Notably, most upregulated miRs are involved in the regulation of monocytic cell differentiation, while most downregulated miRs are implicated in the modulation of T lymphocyte functions.

**Figure 2:**
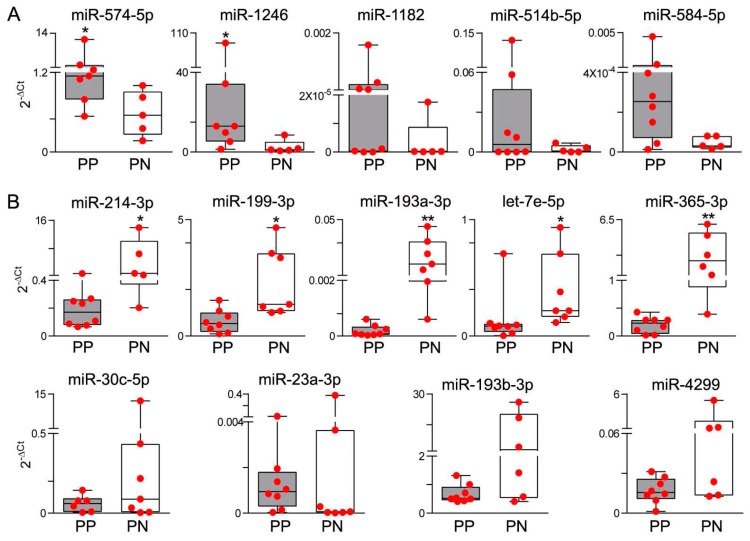
Quantitative real-time PCR (qRT-PCR) analysis confirms differential miR expression patterns in SNB from patients with poor prognosis. Comparative miR expression analysis showing up-regulation of miR-574-5p, miR-1246, miR-1182, miR-514b-5p and miR-584-5p (**A**) and downregulation of miR-214-3p, miR-199-3p, miR-193a-3p, let-7e-5p, miR-365-3p, miR-30c-5p, miR-23a-3p, miR-193b-3p and miR-4299 (**B**) in the PP compared to the PN samples. Twenty-eight SNB samples from patients relapsing (PP, *n* = 16) or not (PN, *n* = 12) within five years of follow-up were tested. The relative quantification was determined using 2^−∆Ct^, and U6 snRNA was used as the internal reference. Statistical analysis was performed by applying the non-parametric Mann-Whitney *U*-test. * *p* < 0.05, ** *p* < 0.01.

**Figure 3:**
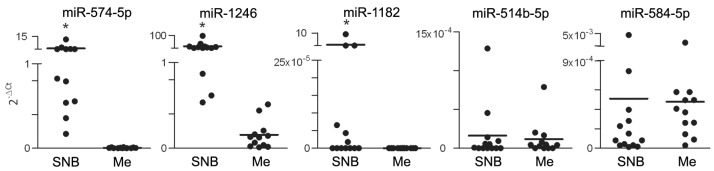
Evaluation of miRs upregulated in SNB from progressing patients in lymph node metastases. Comparative expression analysis of miR-574-5p, miR-1246, miR-1182, miR-514b-5p and miR-584-5p between SNB and massive nodal melanoma metastases (Me, *n* = 12) by qRT-PCR. The relative quantification was determined using 2^−∆Ct^ using U6 snRNA as the internal reference. * *p* < 0.0001 by the non-parametric Mann-Whitney *U*-test.

**Figure 4:**
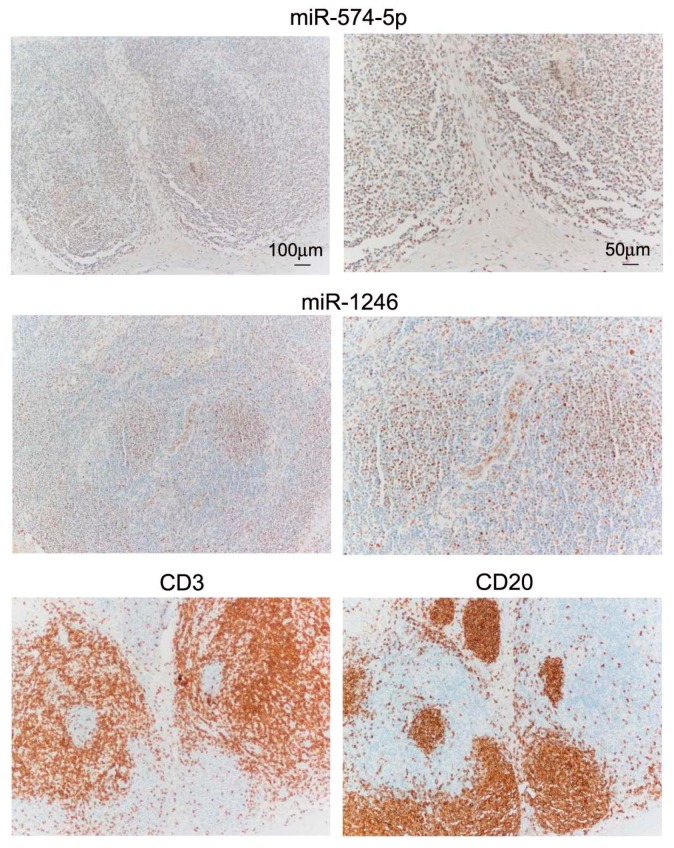
Bright-field in situ hybridization (ISH) detection of miR-574-5p and miR-1246 in SNB. miR-574-5p and miR-1246 signals appear as brown dots localized principally in the nucleus. Both miRs localize in the cortical follicles, and in T and B cells areas as shown by CD3 and CD20 immunostaining in serial sections of the same tissue region. Scale bar is indicated.

Differential expression was tested by qRT-PCR in tumor-positive SNB samples (*n* = 28) and confirmed upregulation of miR-574-5p and miR-1246, and downregulation of miR-214-3p, miR-199-3p, miR-193a-3p, let-7e-5p and miR-365-3p in samples from progressing patients. Other miRs, including miR-1182, miR-514b-5p, miR-584-5p, miR-30c-5p, miR-23a-3p, miR-193b-3p and miR-4299 displayed expression patterns consistent with the results of profiling, although the difference between SNB samples from progressing and non-progressing groups did not reach statistical significance (Figure 2). When the group of SNB from progressing patients was considered, no significant differences in miR expression patterns were observed in SNB from patients with locoregional relapse or metastatic relapse. We have previously ruled out that the different gene expression profiles in SNB could result from varying tumor burden in the lymph nodes. To definitively exclude the potential contribution of infiltrating melanoma cells in determining different levels of expression in SNB samples from progressing patients, upregulated miRs were assessed in the SNB in comparison to melanoma specimens from massive lymph node metastases. Although miR-574-5p and miR-1246 were detectable in all metastases, both miRs showed higher expression levels in SNB. miR-514b-5p and miR-584-5p resulted expressed at similar levels in SNB and in metastases, but they were detected in the majority of SNB and in 9/12 and 3/12 tumor samples respectively; miR-1182 was weakly expressed in SNB and was undetectable in metastases (Figure 3). These data indicated that the expression levels of these miRs in the SNB are not contingent on tumor metastases. To further evaluate miR expression in the sentinel nodes, miR-574-5p and miR-1246, showing the highest expression levels, were evaluated in SNB tissue sections by bright-field in situ hybridization (ISH). Positive staining signals for both miRs were identified as brown dots in the nucleus and, to a lesser extent, in the cytoplasm of cells mostly localized in the cortical region and in the germinal follicles of the node, especially in the T cell areas as shown by the staining with anti-CD3 and CD20 antibodies for T and B cells (Figure 4).

Taken together, these results indicate that the increased miR expression in the SNB from relapsing patients is not ascribable to the infiltrating metastatic melanoma cells, and corroborate our hypothesis that differential miR expression is reasonably linked to a different status of immune cells in the tumor-draining node.

### 3.2. Integrative Analysis of miR and Gene Expression Data Identifies Regulatory Networks in the Lymph Node

**Figure 5:**
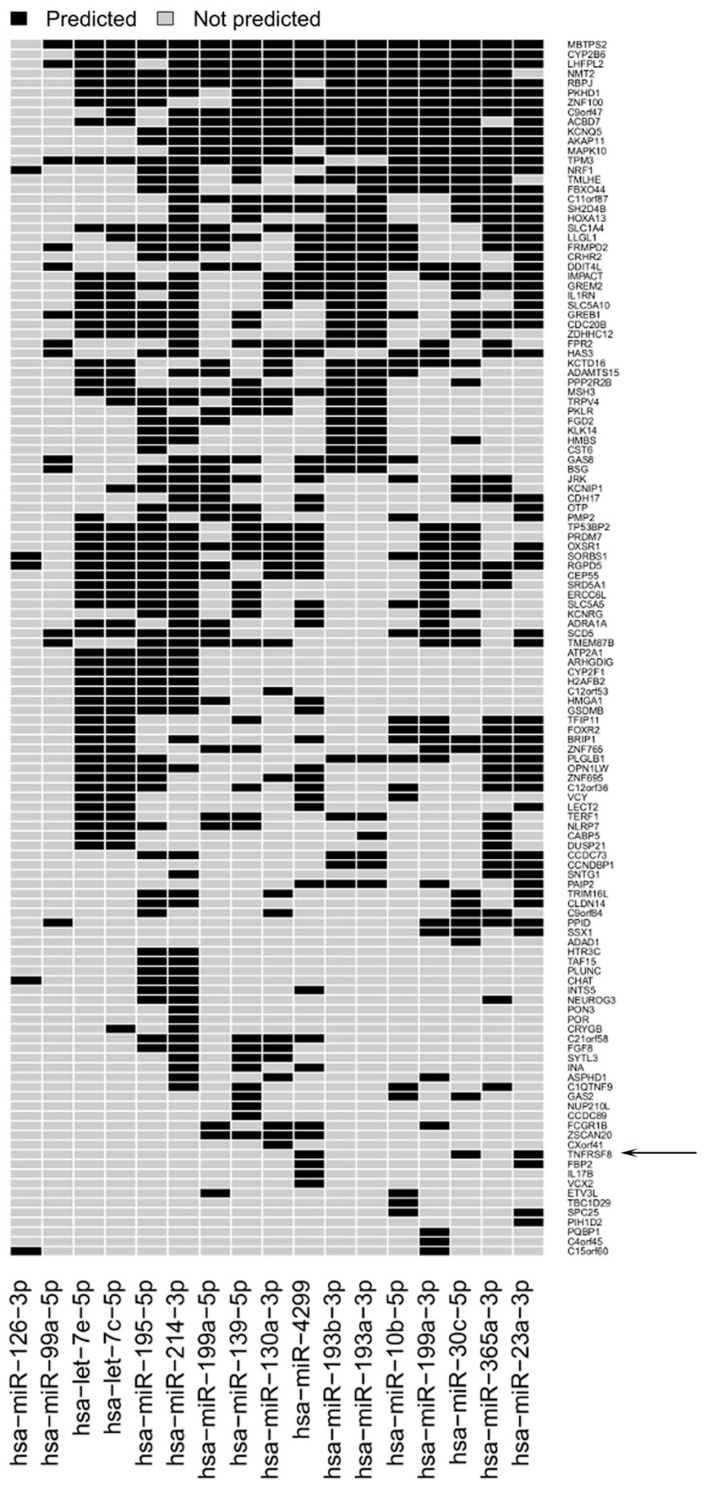
Target prediction of the identified miRs. Heatmap showing predicted target genes for the 17 downregulated miRs in PP versus PN samples. The TNF receptor superfamily member 8 gene (*TNFRSF8*) encoding the CD30 receptor is a predicted target of miR-30c-5p, miR-23a-3p and miR-4299 (indicated by the arrow).

**Figure 6:**
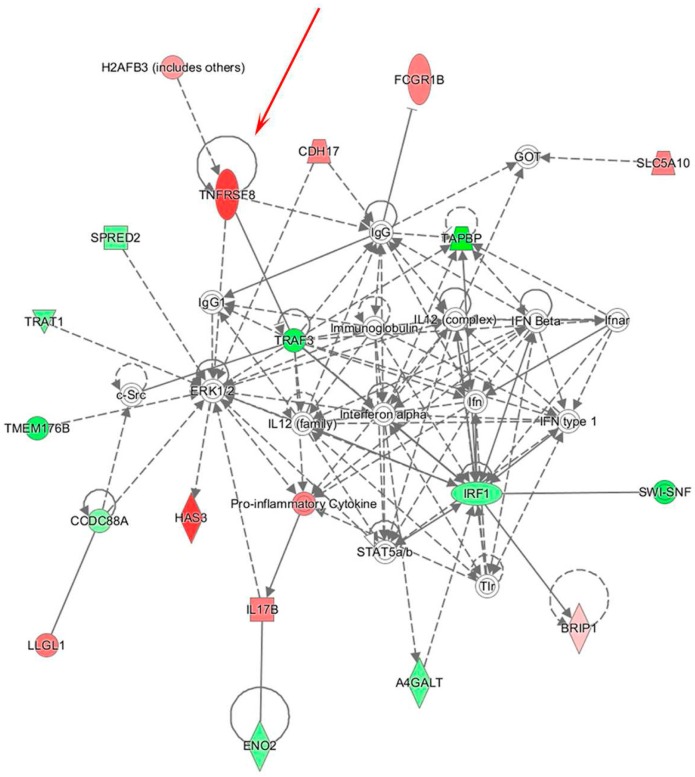
Integrated data analysis reveals a gene network including TNFRSF8/CD30 gene. The “cell-mediated immune response” network identified by IPA was strongly enriched in the list of predicted target genes of the 26 miRs differentially expressed in PP compared to PN samples. The TNFRSF8 gene is indicated by the red arrow.

In order to study potential regulatory mechanisms involving the identified miRs that are associated with immune dysfunction in the sentinel nodes of relapsing patients, combined analysis of miR and gene expression profiles was carried out. Gene expression analysis of the same SNB sample set identified a list of 333 genes differentially expressed between SNB from patients with or without disease progression [8]. The analysis of target prediction performed by applying three different algorithms (Targetscan, microRNA.org database, microT-CDS) and using genes and miRs displaying inverse expression patterns, showed that the miRs target 77% of the regulated genes (Figure 5). IPA analysis of this list of genes showed that one of the main gene networks regulated in PP samples is “cell-mediated immune response”, which included *TNFRSF8* gene encoding for the CD30 receptor (Figure 6). CD30 is a receptor expressed at high levels by different types of activated immune cells, and its interaction with the cognate CD30L has a recognized role in autoimmune and chronic inflammatory diseases [36]. Our previous findings revealed a possible role for CD30 in tumor immunity, since higher numbers of CD30-positive lymphocytes representing different populations of suppressive or exhausted immune cells are detected in melanoma patients with progressive disease [8]. The gene network also revealed a possible altered expression of the IFN and IFN-inducible genes in SNB samples, as indicated by the downregulation of the activator of IFN alpha and beta transcription *IRF1* gene, together with a compromised immune response, as indicated by the abovementioned upregulation of *TNFRSF8* gene, paralleled by the downregulation of *TAPBP*, required for optimal peptide loading on the MHC class I molecule, *TRAF3*, which participates in the signal transduction of CD40, *TRAT1*, which stabilizes the TCR/CD3 complex in T cells, and *TMEM176B*, involved in the process of maturation of dendritic cells. These genes resulted as potential targets of all the upregulated miRs. IPA analysis revealed also that 15/17 miRs downregulated in SNB from progressing patients target immune-related genes, resulting upregulated. Besides *TNFRSF8*, other examples include the *IL1RN* gene (modulator of several IL1-related immune and inflammatory responses), the *RBPJ* gene (transcriptional regulator important for B cells), the *FCGR1B* gene (playing a role in humoral immune response) and the *IL17B* gene (cytokine stimulating the release of IL1b and TNFa).

### 3.3. Modulation of miRs during In Vitro Induction of CD30 in Immune Cells

**Figure 7:**
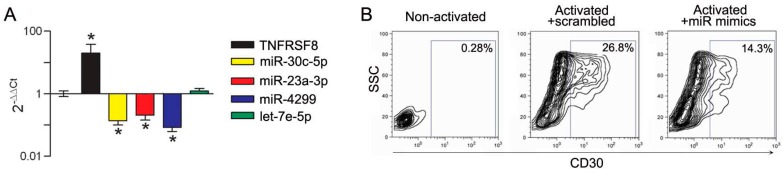
miR-30c-5p, miR-23a-3p and miR-4299 modulate CD30 expression in activated CD4^+^CD25*^−^* effector T cells. (**A**) qRT-PCR analysis showing that CD4^+^CD25*^−^* cells upregulate expression of TNFRSF8 gene and downregulate expression of miR-30c-5p, miR-23a-3p and miR-4299 upon activation, while let-7e-5p expression levels are not modulated. Relative quantification was determined using 2^−∆∆Ct^ method, by using the non-activated cells as the calibrator, while U6 snRNA was used as the internal reference. * *p* < 0.0001 by unpaired *t*-test; (**B**) FACS analysis showing the expression of CD30 in non-activated lymphocytes, and in activated lymphocytes transiently transfected with mimics of miR-30c-5p, miR-23a-3p and miR-4299 or with a scrambled control. The percentage of CD30 positive cells is reported.

The TNFRSF8 gene is a predicted target for miR-30c-5p, miR-23a-3p and miR-4299. To evaluate whether these miRs could be functionally associated with CD30, their expression was assessed by qRT-PCR in immune cells upon in vitro CD30 modulation. We first assessed CD4 lymphocytes induced to express CD30 by TCR engagement, a setting in which CD30 is highly upregulated. In CD4^+^CD25^−^ lymphocytes from healthy donors (HD) stimulated in vitro with anti-CD3/CD28 microbeads, the expression of TNFRSF8/CD30 transcript was upregulated and, concomitantly, miR-30c-5p, miR-23a-3p and miR-4299 were downregulated (Figure 7A). CD30 upregulation was confirmed at protein level by FACS analysis (Figure 7B). Further evidence of the functional association between CD30 and the miRs was obtained by activating CD4^+^CD25^−^ lymphocytes in the presence of miR-30c-5p, miR-23a-3p and miR-4299 overexpression obtained by using specific mimics. Transfection of the mimics, leading to upregulated miR expression levels in CD4 lymphocytes up to 10^4^–10^5^ fold compared to a scrambled control (Supplementary material Figure S1), was associated with a significant reduction of CD30 expression as evaluated by FACS analysis (Figure 7B). These results clearly indicate that the identified miRs play a role in regulating CD30 expression in CD4 effector cells.

**Figure 8:**
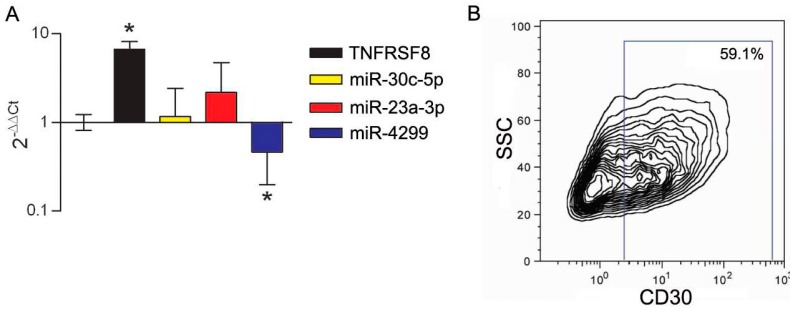
miR-4299 downmodulation is associated with CD30 induction in activated Tregs. (**A**) Tregs upregulate the expression of the TNFRSF8 gene and downregulate the expression of miR-4299 upon stimulation, as determined by qRT-PCR analysis. The 2^−∆∆Ct^ quantification was determined using non-activated CD4^+^CD25^−^ as the calibrator, while U6 snRNA was used as the internal reference. * *p* < 0.0001 by unpaired *t*-test; (**B**) Expression of CD30 is induced in purified Tregs upon in vitro activation as shown by FACS analysis.

**Figure 9:**
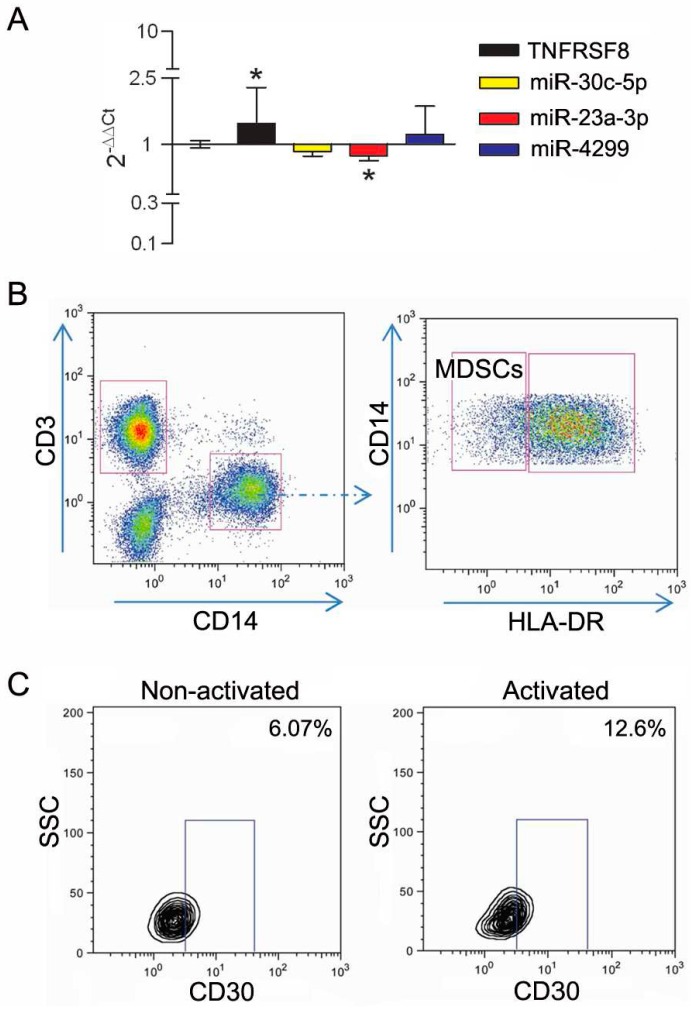
miR-23a-3p downmodulation is associated with upregulated CD30 expression in myeloid-derived suppressor cells (MDSCs). (**A**) qRT-PCR analysis showing that peripheral blood mononuclear cells (PBMCs) stimulated with melanoma-conditioned medium upregulate expression of TNFRSF8 and downregulate miR-23a-3p. The 2^−∆∆Ct^ quantification was determined using non stimulated PBMCs as the calibrator, while U6 snRNA was used as the internal reference. * *p* < 0.05 by unpaired *t*-test; (**B**) Gating strategy for MDSC identification: MDSCs were identified inside live cells as CD14^+^CD3^−^ with a low/negative expression of HLA-DR; (**C**) Contour plots show the expression of CD30 in MDSCs. Percentage of positive cells, based on isotype IgG control, is indicated for non-activated and for activated lymphocytes.

We previously showed that CD30 displays enhanced expression in immune cells with suppressive activity in the melanoma-invaded lymph node [8]. We thus tested CD30 upregulation upon activation of CD4^+^CD25^+^ purified Tregs. In this setting, the upregulation of the CD30 transcript and protein was accompanied by the selective downregulation of miR-4299 (Figure 8). In addition, to mimic in vitro the influence of melanoma-released factors on CD30 expression, PBMCs cells from HD were cultured in the presence of melanoma conditioned medium. In this setting, the expression of CD30 was upregulated and a coordinated significant downregulation was detected only for miR-23a-3p. Interestingly, FACS analysis showed that, upon stimulation with melanoma-derived factors, CD30 was selectively upregulated in the CD14^+^DR^−^ monocyte population (Figure 9), a cell subset known as monocytic myeloid-derived suppressor cells (MDSCs) [37].

In conclusion, these results indicated that miR-30c-5p, miR-23a-3p and miR-4299 are involved in the regulation of CD30 expression, and that miR regulation of CD30 induction is cell population specific.

## 4. Discussion

There is compelling evidence that in melanoma the interactions between tumor and the immune system impact patient outcome. Tumor core infiltration of CD8 lymphocytes in primary tumors and in nodal metastases positively correlates with survival [38,39]. Immune-related gene expression profiles in primary tumors and in regional metastases are positively associated with prognosis [40,41]. Confirmation is also emerging in the setting of response to immunotherapy and to targeted drugs, indicating that immune signatures may be predictive of response to treatment [42,43].

Few data are available on the prognostic value of the immunological status of the sentinel node, although several authors reported immunosuppressive changes occurring in the tumor-draining nodes [44,45,46]. We have recently observed that the transcriptional profiles of SNB of stage III patients distinguish melanoma patients with progressive disease [8], suggesting that the immunological setting in the SNB may anticipate the conditions of systemic immunity and provide prognostic information. To the best of our knowledge, this is the first study to analyze miR profiles in SNB samples. miRs have recognized pivotal roles in the regulation of the physiological functions of immune cells, and their expression is dysregulated in pathological conditions involving the immune system, such as autoimmunity and cancer [33]. In fact, miRs regulate the functions of major protumorigenic immune subpopulations involved in cancer immune evasion, MDSCs and Tregs [15,47]. Our results show that most miRs regulated in SNB are involved in the regulation of monocytic differentiation and of T lymphocytes functions. Notably, several miRs identified here were also found by sequencing in a single SNB of a melanoma patient in the pioneering study by Ma and collaborators [48]. Among the miRs upregulated in SNB from patients with disease progression, miR-574-5p and miR-1246 were shown as expressed in different lymphocytes populations in the lymph node by bright-field ISH, especially but not exclusively in the cortical region. Notably, miR-1246 takes part in the miR signature reported to characterize the Treg phenotype [16], while miR-574-5p is involved in the differentiation of monocytes [15]. miR-1246 is released by different types of cells in vitro, including monocytes upon differentiation to M1 macrophages [49]. Notably, circulating miR-1246 is reported to be a potential biomarker for different tumor types, including metastatic melanoma [50].

In order to identify in our setting regulatory mechanisms associated with immune dysfunction involving the identified miRs, we carried out analysis of target prediction by different algorithms considering the differentially expressed genes identified in the same samples in a previous study [8]. The analysis by IPA of the genes potentially targeted by the identified miRs on the basis of sequence complementarity identified a gene network involving *TNFRSF8*/CD30 and other genes potentially contributing to defective tumor immunity. As our published and unpublished data indicate that the CD30 receptor is a potential player in tumor-induced immunosuppression, we focused our analysis on three miRs, namely miR-30c-5p, miR-23a-3p and miR-4299, which were predicted to target the *TNFRSF8*/CD30 gene as well as other genes in the gene network. Analysis of coordinated and inverse *TNFRSF8*/CD30 and miR-30c-5p, miR-23a-3p and miR-4299 expression was carried in different lymphocyte settings during CD30 in vitro induction. In particular, Tregs and MDSCs, two key members of cancer-associated immunosuppression, were found to upregulate CD30, likely through selective modulation by miR-30c-5p, miR-23a-3p and miR-4299. We observed a cell type-dependent variation in miR expression upon CD30 induction, between CD4 effector and suppressive lymphocytes, and between CD14^+^DR^−^ monocytes and Tregs, suggesting that miR involvement is cell type-specific. miR-30c-5p, miR-23a-3p and miR-4299 were previously reported to affect differentiation of DCs and of monocytes [25] and to repress cytotoxicity of T cells [28], but their role in Tregs has not yet been reported. miRs potentially associated with systemic immune suppression can be further exploited in studies considering their possible targeting for counteracting disease progression.

## 5. Conclusions

So far, molecular analysis in SNB has been studied with the purpose of increasing the detection of metastatic melanoma cells, generally by testing melanoma markers by qRT-PCR [51]. Although the detection of these markers improved the rate of positive samples, it is still unclear whether positive detection has a prognostic significance. Conversely, the results of our pilot study foster the idea that immune molecular markers in SNB may hold information to identify patients at high risk for disease progression that would potentially benefit of adjuvant treatments. Molecular signatures including miRs potentially indicating the risk of relapse over the current American Joint Committee on Cancer TNM (Tumor, Nodes, Metastasis) staging system for stage III patients are currently being studied in our laboratory in a larger study for validation and eventually for inclusion in risk assessment.

## Figures and Tables

**Table 1:** miRs differentially expressed between tumor-positive SNB from patients relapsing or not at five years of follow-up.

Gene Name	Fold Change	FDR	*p*-Value
miR-574-5p	2.237	0.066	0.001
miR-1182	1.393	0.066	0.001
miR-1246	1.393	0.066	0.002
miR-514b-5p	1.342	0.072	0.004
miR-424-3p	1.336	0.067	0.003
miR-3147	1.326	0.066	0.002
miR-765	1.324	0.088	0.005
miR-1306-3p	1.272	0.072	0.003
miR-584-5p	1.238	0.066	0.001
miR-214-3p	−2.209	0.066	0.001
miR-199a-3p ^a^	−1.731	0.066	0.002
miR-130a-3p	−1.711	0.045	0.000
miR-195-5p	−1.699	0.066	0.002
miR-30c-5p	−1.622	0.066	0.003
miR-193a-3p	−1.592	0.066	0.002
let-7c-5p	−1.572	0.072	0.003
miR-23a-3p	−1.559	0.080	0.005
miR-99a-5p	−1.552	0.075	0.004
miR-199a-5p	−1.541	0.066	0.001
miR-126-3p	−1.539	0.045	0.000
miR-193b-3p	−1.519	0.066	0.003
let-7e-5p	−1.512	0.088	0.005
miR-365a-3p ^b^	−1.503	0.066	0.001
miR-10b-5p	−1.500	0.066	0.002
miR-4299	−1.490	0.066	0.002
miR-139-5p	−1.410	0.080	0.004

^a^ Probe sequence (TAACCAATGTGCAGACTACT) of miR-199a-3p (MIMAT0000232) detects also miR-199b-3p (MIMAT0004563); ^b^ Probe sequence (ATAAGGATTTTTAGGGGCATTA) of miR-365a-3p (MIMAT0000710) detects also miR-365b-3p (MIMAT0022834); FDR: false discovery rate.

**Table 2:** Functional involvement of the differentially expressed miRs in immune cells.

Gene Name	Regulation	Immune Compartment	Functions in Immune System	Reference
miR-574-5p	↑	myeloid	promotes differentiation in monocytes	[15]
miR-1246	↑	T lymphocytes	upregulated in Tregs	[16]
miR-424-3p	↑	myeloid	involved in monocyte differentiation	[17,18]
miR-584-5p	↑	myeloid	regulated in AML CD34^+^ cell line	[19]
miR-214-3p	↓	T lymphocytes	upregulated in stimulated T cells and Tregs	[20,21]
miR-199a-3p	↓	myeloid	involved in leukemogenesis	[22]
miR-130a-3p	↓	T lymphocytes	upregulated in CD8 T cells inhibits CD69	[23]
miR-195-5p	↓	myeloid	regulated in monocytes	[24]
miR-30c-5p	↓	myeloid	regulated in mDCs and pDCs	[25]
let-7c-5p	↓	myeloid	promotes M2 polarization	[26,27]
miR-23a-3p	↓	T lymphocytes; myeloid	represses CTL activity; regulated in mDCs	[25,28]
miR-99a-5p	↓	B lymphocytes	involved in B cell differentiation	[29]
miR-199a-5p	↓	T lymphocytes	overexpressed in T cells vs ALCL ALK^+^	[30]
miR-126-3p	↓	T lymphocytes	downregulated in activated T cells	[31]
miR-193b-3p	↓	T lymphocytes	involved in T cell maturation	[32]
let-7e-5p	↓	myeloid	regulated in macrophage upon TLR4	[33]
miR-365a-3p	↓	T lymphocytes	upregulated in T activation	[31]
miR-10b-5p	↓	myeloid	upregulated in CD34^+^ in myelofibrosis	[34]
miR-139-5p	↓	T lymphocytes	regulated in CTLs	[35]

Expression pattern in SNB from patients with progressing disease: ↑, increased expression; ↓, decreased expression. Tregs: regulatory T lymphocytes; AML: acute myeloid leukemia; mDCs: monocyte-derived dendritic cells; pDCs: plasmacytoid dendritic cells; CTLs: cytotoxic T lymphocytes; ALCL: anaplastic large cell lymphoma; ALK: anaplastic lymphoma kinase; MDSCs: myeloid-derived suppressor cells; TLR4: Toll-like receptor 4.

## Supplementary Materials

The following are available online at www.mdpi.com/2073-4425/7/12/124/s1, Table S1: Clinicopathological data for the sentinel node biopsy (SNB) samples analyzed in the study, Figure S1: Overexpression of miR-30c-5p, miR-23a-3p and miR-4299 after transfection with miR mimics.
