# Supplementary material for: microRNA Expression in Sentinel Nodes from Progressing Melanoma Patients Identifies Networks Associated with Dysfunctional Immune Response
Source: Genes (Basel). 2016 Dec 14;7(12):124. doi: 10.3390/genes7120124 (PMC5192500; doi:10.3390/genes7120124)
Supplement: Supplementary file 1 [file genes-07-00124-s001.docx]

Article

microRNA Expression in Sentinel Nodes from Progressing Melanoma Patients Identifies Networks Associated with Dysfunctional Immune Response

Viviana Vallacchi, Chiara Camisaschi, Matteo Dugo, Elisabetta Vergani, Paola Deho, Ambra Gualeni, Veronica Huber, Annunziata Gloghini, Andrea Maurichi, Mario Santinami, Marialuisa Sensi, Chiara Castelli, Licia Rivoltini, Monica Rodolfo

**Table S1.** Clinicopathological data for the sentinel node biopsy (SNB) samples analyzed in the study

| **ID** | **SNB Status ^a^** | **Patient status at 5y FU ^b^** | **SNB Tumor Burden at Histopathology ^c^** | **Stage after Surgery ^d^** | **Primary Tumor Classification** | **Primary Tumor Localization ^e^** | **Gender** | **Age** | **Relapse LR/MET ^f^** | **Tested for ^g^** |
| --- | --- | --- | --- | --- | --- | --- | --- | --- | --- | --- |
| LN7 | neg | N | neg | IB | T1b | L | F | 61 |  | A |
| LN12 | neg | N | neg | IIB | T3b | L | F | 30 |  | A |
| LN1 | neg | N | neg | IIA | T3a | L | F | 41 |  | A |
| LN11 | neg | N | neg | IB | T1b | L | M | 57 |  | A |
| LN10 | neg | N | neg | IB | T2b | F | F | 51 |  | A |
| LN17 | neg | N | neg | IB | T1b | T | M | 41 |  | A |
| LN22 | neg | N | neg | IB | T1b | F | F | 62 |  | A |
| LN8 | neg | N | neg | IB | T2a | L | M | 52 |  | A |
| LN3 | pos | PN | 6, m, 1.8, ip | IIIA | T3a | F | F | 69 |  | A+B |
| LN6 | pos | PN | single cells, ip | IIIA | T3a | F | F | 55 |  | A |
| LN9 | pos | PN | 3, M, 2.88, ip | IIIB | T3b | L | F | 52 |  | A+B |
| LN13 | pos | PN | 3, m, sc + ip | IIIA | T2b | T | M | 46 |  | A+B |
| LN14 | pos | PN | 2, m, ip | IIIA | T3a | L | F | 74 |  | A+B |
| LN15 | pos | PN | 1, m, ip | IIIA | T3b | F | F | 38 |  | A |
| LN19 | pos | PN | 1, m, sc | IIIA | T2a | F | F | 51 |  | A+B |
| LN49 | pos | PN | 6, M, 1.8, ip | IIIB | T3a | F | F | 70 |  | A |
| LN24 | pos | PP | 4, M, ip | IIIC | T2b | T | F | 58 | MET | A+B |
| LN23 | pos | PP | 2, m, sc | IIIB | T2b | F | F | 76 | LR | A+B |
| LN4 | pos | PP | 1, 2.1, ip | IIIC | T3b | F | F | 73 | LR | A+B |
| LN2 | pos | PP | 1, M, ip | IIIC | T3b | L | M | 68 | LR | A+B |
| LN21 | pos | PP | 1, M, ip | IIIC | T3b | L | M | 66 | MET | A+B |
| LN16 | pos | PP | 2, M, ip | IIIC | T3a | T | F | 57 | MET | A+B |
| LN18 | pos | PP | 4, M, ip | IIIC | T3b | L | M | 69 | MET | A+B |
| LN5 | pos | PP | 1, m, ip | IIIC | T2a | F | M | 67 | MET | A+B |
| LN53 | pos | PN | 3,m,0.36,sc + ip | IIIB | T3a | L | M | 46 |  | B |
| LN54 | pos | PN | 3,m, ip | IIIA | T3b | F | F | 74 |  | B |
| LN65 | pos | PN | 2, M, 3.5, ip | IIIA | T2a | T | F | 54 |  | B |
| LN66 | pos | PN | single cells, ip | IIIA | T3a | F | F | 55 |  | B |
| LN50 | pos | PN | 3,m, ip | IIIA | T3A | T | F | 54 |  | B |
| LN51 | pos | PN | 2, M, 3.5, ip | IIIB | T3B | L | F | 53 |  | B |
| LN52 | pos | PN | single cells, ip | IIIA | T2A | T | F | 51 |  | B |
| LN37 | pos | PP | 5, M, 2.4, sc | IIIB | T4b | L | F | 71 | MET | B |
| LN39 | pos | PP | 3, M, 2.6, ip + ex | IIIC | T4b | F | M | 73 | MET | B |
| LN44 | pos | PP | single cells, ip | IIIB | T1b | L | F | 65 | MET | B |
| LN47 | pos | PP | 1, M, ip | IIIC | T4b | T | F | 29 | MET | B |
| LN64 | pos | PP | 1, M, ip | IIIC | na | L | F | 46 | LR | B |
| LN41 | pos | PP | 2,M,0.8, ip,ex | IIIB | T4B | L | M | 52 | LR | B |
| LN42 | pos | PP | single cells, ip | IIIB | T4B | L | F | 65 | LR | B |
| LN43 | pos | PP | single cells, ip | IIIB | T1B | T | F | 49 | LR | B |

a. status of sentinel nodes relative to melanoma infiltration, pos: positive for metastases, neg: negative for metastases; b. disease course in 5 years of follow-up, N: negative, PN: not progressing, PP: progressive disease; c. hystopathological evaluation of tumor burden; number of metastatic foci; size of metastases, m: <1 mm, M: >1mm, larger diameter of the larger focus in mm; localization of metastases, ip: intraparenchimal, sc: subcapsular, ex: extracapsular; d. pathologic stage after complete lymph node dissection (CLND) in SNB-positive patients; e. L: leg, F: foot, T: trunk. All SNB samples were inguinal; f. LR: locoregional relapse, MET: metastatic relapse;
g. A: tested by microRNA (miR) arrays; B: tested by quantitative real-time PCR *(*qRT-PCR); na: not available.


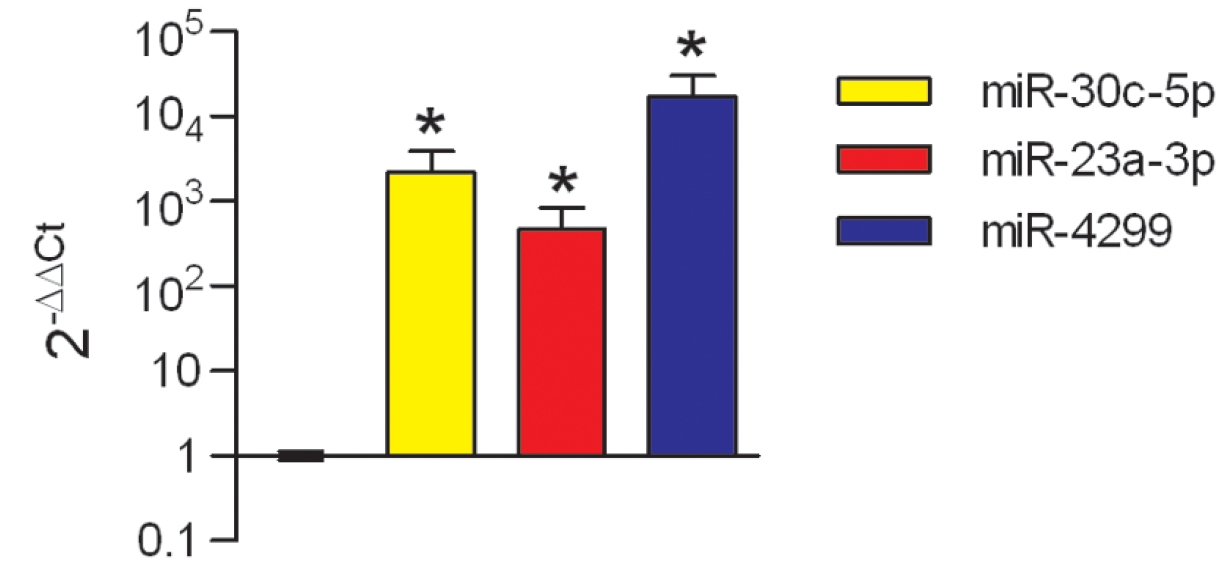


**Figure S1.** Overexpression of miR-30c-5p, miR-23a-3p and miR-4299 after transfection with miR mimics. qRT-PCR analysis was performed in CD4^+^CD25^−^ cells 72 h after transfection with miRNA mimics or a scrambled control. The relative quantification was determined using 2^−∆∆Ct^ method, using cells transfected with scrambled control as calibrator. U6 snRNA was used as the internal reference.
* *p* < 0.0001 by unpaired *t*-test.

© 2016 by the authors. Submitted for possible open access publication under the
terms and conditions of the Creative Commons Attribution (CC-BY) license (http://creativecommons.org/licenses/by/4.0/).
